# Aberrant nuclear localization of β-catenin without genetic alterations in β-catenin or Axin genes in esophageal cancer

**DOI:** 10.1186/1477-7819-5-21

**Published:** 2007-02-19

**Authors:** Junzo Kudo, Tadashi Nishiwaki, Nobuhiro Haruki, Hideyuki Ishiguro, Yasuyuki Shibata, Yukio Terashita, Hironori Sugiura, Noriyuki Shinoda, Masahiro Kimura, Yoshiyuki Kuwabara, Yoshitaka Fujii

**Affiliations:** 1 Department of Surgery II, Nagoya City University Medical School, Nagoya, Japan

## Abstract

**Background:**

β-catenin is a multifunctional protein involved in two apparently independent processes: cell-cell adhesion and signal transduction. β-catenin is involved in Wnt signaling pathway that regulates cellular differentiation and proliferation. In this study, we investigated the expression pattern of β-catenin and cyclin D1 using immunohistochemistry and searched for mutations in exon 3 of the β-catenin gene and Axin gene in esophageal squamous cell carcinoma.

**Materials and methods:**

Samples were obtained from 50 esophageal cancer patients. Immunohistochemical staining for β-catenin and cyclin D1 was done. Mutational analyses of the exon3 of the β-catenin gene and Axin gene were performed on tumors with nuclear β-catenin expression.

**Results:**

Four (8%) esophageal cancer tissues showed high nuclear β-catenin staining. Overexpression of cyclin D1 was observed in 27 out of 50 (54%) patients. All four cases that showed nuclear β-catenin staining overexpressed cyclin D1. No relationship was observed between the expression pattern of β-catenin and cyclin D1 and age, sex, tumor size, stage, differentiation grade, lymph node metastasis, response to chemotherapy, or survival. No mutational change was found in β-*catenin *exon 3 in the four cases with nuclear β-catenin staining. Sequencing analysis of the *Axin *cDNA revealed only a splicing variant (108 bp deletion, position 2302–2409) which was present in the paired normal mucosa.

**Conclusion:**

A fraction of esophageal squamous cell carcinomas have abnormal nuclear accumulation of β-catenin accompanied with increased cyclin D1 expression. Mutations in β-catenin or axin genes are not responsible for this abnormal localization of β-catenin.

## Background

β-catenin is a multifunctional protein involved in two apparently independent processes: cell-cell adhesion and signal transduction. β-catenin binds to both the cytoplasmic domain of cadherin and the amino-terminal domain of β-catenin and mediates cell adhesion. In addition to its function in cell-cell adhesion, β-catenin plays an important role in signal transduction; it is involved in the Wnt signaling pathway that regulates cellular differentiation and proliferation [1].

The level of free β-catenin is low in normal cells, since the protein is sequestered in a complex, which includes the adenomatous polyposis coli (APC) protein, a serine threonine glycogen kinase (GSK-3β) and conductin or Axin, leading to degradation of β-catenin by proteasome. The binding of β-catenin by APC requires phosphorylation of β-catenin by GSK-3β on 3 serine and 1 threonine residues, all of which are encoded by exon 3 of the β-*catenin *gene [2-4].

In colorectal cancers, mutations of APC or β-catenin result in stabilization of β-catenin and a significant accumulation of this protein within the cytoplasm [5]. Furthermore, increased β-catenin may translocate to the nuclei and could serve as a transcriptional factor by binding to the T-cell factor/lymphoid enhancing factor (Tcf-Lef) family [5], leading to transcription of specific genes stimulating tumor formation, such as *cyclin-D1, c-myc, c-jun, fra-1, uPAR, ZO-1, MMP7, NBL4, DRCTNNB1A, MCP-3 *[6-12]. However, the precise regulatory mechanisms remain to be resolved. Mutations, including large interstitial deletions involving exon 3 of the β-catenin gene, have also been found in several other tumors [5,13,14].

Recently, cyclin D1 has been identified as a target of the β-catenin/T-cell factor/lymphoid enhancer factor complex [2,15]. Cyclin D1 is expressed in the G1 phase of the cell cycle, and is thought to play a major role in the control of the cell cycle and cancer progression. Overexpression of cyclinD1 has been suggested to contribute to oncogenesis by disturbing the cell cycle and has been reported to be an important oncogenic factor in esophageal carcinoma [16].

Recent experiments demonstrated that Axin functions as a tumor suppressor in hepatocellular carcinoma [17]. The different domains of Axin possess binding capacity for APC, GSK-3β, β-catenin, PP2A, Dishevelled, and Axin itself [18,19]. As a scaffold protein of this multiprotein complex, Axin is able to bring β-catenin and GSK-3β into close proximity, thus facilitating β-catenin phosphorylation [20,21] and subsequent ubiquitin-mediated degradation by the proteasome system [22,23].

Esophageal squamous cell carcinoma is an aggressive disease with a poor prognosis, and the genetic mechanism of its carcinogenesis remains to be solved. The progression of this tumor is associated with multiple genetic alterations, including loss of heterozygosity in chromosomes 3p, 5q, 9p, 9q, 13q, 17p, 17q and 18q, and amplification of epidermal growth factor receptor (EGFR), HER-2, c-myc, and cyclin D1 [24]. The most frequent genetic alteration in esophageal squamous cell carcinoma is a point mutation in the p53 gene (40–60%) that occurs at a relatively early stage of tumor development [13,25]. However, Wnt signal pathway in esophageal cancer has not been extensively studied.

In this paper, we investigated the expression pattern of β-catenin and cyclin D1 in esophageal squamous cell carcinoma. We found aberrant localization of β-catenin in a minor proportion of the tumor and mutation in exon 3 of the β-*catenin *gene (CTNNB1) and Axin gene was studied in these samples.

## Materials and methods

### Tissue samples

Samples were obtained from 50 esophageal cancer patients who had undergone operations at the Department Surgery II, Nagoya City University Medical School between 1996 and 2000. They were classified according to the guideline for the clinical and pathologic studies on carcinoma of the esophagus [26]. All samples for RT-PCR were frozen immediately in liquid nitrogen and stored -80°C until analysis. All the tissues for immunohistochemistry was fixed in formalin and embedded in paraffin.

### Immunohistochemical staining for β-catenin and cyclin D1

Immunohistochemical staining was done with anti-β-catenin monoclonal antibody (Transduction Lab.) and anti-cyclin D1 monoclonal antibody (Oncogene Research Product). A formalin-fixed paraffin-embedded tissue block was cut and de-waxed. Each section was treated for 15 min in citric-acid buffer (pH 6.0) with autoclave at 120°C for antigen retrieval, and was immunostained with the standard indirect ABC technique. Counterstaining was done with hematoxylin. When more than 20% of the cells were stained, the staining was scored as positive.

### RNA extraction and reverse transcriptional reaction

Total RNA was extracted from the esophageal cancer tissue, and normal esophageal mucosa taken from the mucosa as far apart from the tumor as possible, using Isogen kit (Nippon gene, Tokyo, Japan). The concentration of the RNA was adjusted to 200 ng/ml, using spectrophotometer. Reverse transcriptional reaction was carried out at 42°C for 90 min and 95°C for 5 min followed by incubation at 72°C for 15 min, using 1 μg RNA, 0.5 mg oligo(dTi) primer, Superscript II enzyme (Gibco BRL, Gaithersburg, MD).

### Mutational analyses of the exon3 of the β-catenin and axin genes

Polymerase chain reaction (PCR) and direct sequencing analysis were performed on the four tumors with nuclear β-catenin expression. Four microliter of cDNA mixture was subjected to amplification in 80 μl containing 0.8 U of Taq polymerase, 10×PCR buffer, 25 μM each of dATP, dCTP, dGTP, dTTP, 25 μM of MgCl2 and 80 pmol each of 5' and 3' primers. PCR conditions for β-catenin were initial denaturation for 5 min at 95°C, followed by 38 cycles of denaturation at 94°C for 30 sec, annealing at 56°C for 30 sec, extension at 72°C for 30 sec and a final extension at 72°C for 5 min. PCR conditions for axin were initial denaturation for 5 min at 95°C, followed by 38 cycles of denaturation at 94°C for 60 sec, annealing at 54°C for 60 sec, extension at 72°C for 60 sec and a final extension at 72°C for 5 min. The primer pair used for amplification of the entire exon 3 of the β-catenin gene and four sets of primers for axin gene are shown in the Table 1. The identities of PCR products were analyzed by a 3% agarose gel electrophoresis. After electrophoresis, the PCR products were purified from agarose gels using a QIAquick gel extraction kit (Qiagen, Hilden, Germany) and amplified with the primer set shown in Table 1. Sequencing was performed with the ABI 3100 DNA Sequencer (Applied Biosystems, Foster City, Calif.).

**Table 1:** Primer sequences and annealing temperatures for PCR

Primer set	Position	Annealing temperature
β-catenin exon3		
F:GTC TGA GGA GCA GCT TCA GT	77–96	56°C
R:CAT TAG TGG GAT GAG CAG CA	559–578	
Axin		
F1:GCG GGA CAG ATT GAT TCA CT	28–47	54°C
R1:TCG GCA GGT ATC CAG ATA TG	811–830	
F2:TCT GTA GTG ACC AGA GCT CT	771–790	54°C
R2:GAC GAT GGA TCG CCG TCC T	1380–1398	
F3:AGT TCG CGG AGG AGC TCA T	1272–1290	54°C
R3:CCT CAA TGA TCC ACT GCA TG	2011–2030	
F4:AGG ATG CGG AGA AGA ACC AG	1986–2005	54°C
R4:TCC TGA GTA CGA GGT CAT CT	2770–2789	

### Statistical method

χ-square test was used to analyze the relationship between the two variables. A p value of less than 0.05 was considered significant.

## Results

### Immunohistochemical study for β-catenin

**Figure 1:**
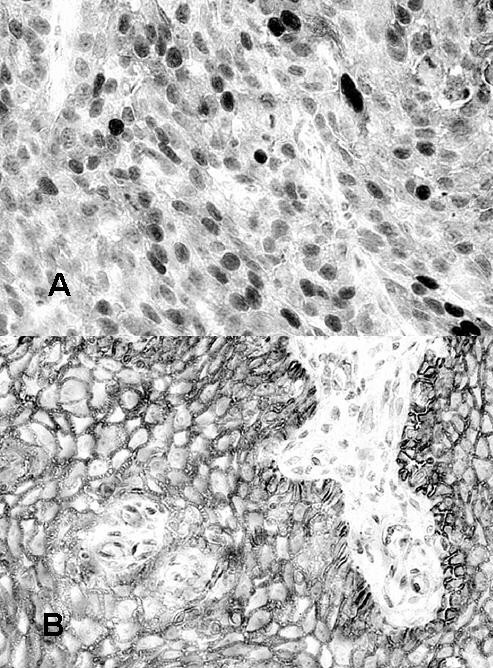
Immunohistochemical staining of β-catenin in normal and cancer tissues of the esophagus. In this cancer tissues, β-catenin was expressed in the nuclei, and was hardly detectable at the plasma membrane (a). In normal esophageal epithelium, β-catenin was expressed at the cell membrane (b).

Fifty esophageal cancer tissues and paired normal esophageal mucosa were stained for β-catenin using immunohistochemistry. In all the normal esophageal mucosa, β-catenin staining was restricted to the plasma membrane. The intensity of the staining did not vary very much among individuals. In most of the 50 esophageal cancer tissues, the β-catenin staining was at the plasma membrane. However, four cases (8%) of esophageal cancer tissue were judged as showing widespread nuclear staining. And in these cases, β-catenin was not expressed on the plasma membrane (Fig. 1a). Normal esophageal mucosa of these cases showed membrane staining of β-catenin, and did not show nuclear staining (Fig. 1b). 46 other esophageal cancer samples showed either plasma membrane or cytoplasmic staining or both. No relationship was observed between β-catenin expression pattern and age, sex, tumor size, stage, differentiation grade, lymph node metastasis, response to chemotherapy, or survival (χ-square test, Table 2).

**Table 2:** Clinico-pathological characteristics and β-catenin and cyclin-D1 protein expression in 50 patients with esophageal cancer

	age/sex	follow-up(M)	out come^a)^	T	N	stage	ly^b)^	v^c)^	diff^d)^	β-catenin^e)^	cyclin D1
1	67M	31	D.O	1	0	I	0	0	M	M	+
2	46M	24	A	1	0	I	0	0	M	C	-
3	68M	26	A	1	1	I	1	1	W	N+C+M	+
4	56M	11	A	1	0	I	0	0	M	M	-
5	68M	18	A	1	0	I	0	0	M	M	+
6	66M	36	D.E	3	0	II	2	1	M	C+M	-
7	73F	38	A	2	1	II	1	0	W	M	-
8	69M	2	D.O	1	2	II	0	0	W	M	-
9	60M	32	A	1	2	II	0	0	W	M	+
10	58M	16	D.E	1	1	II	1	1	P	N+C	+
11	79F	30	D.E	3	0	III	1	1	W	N+C	+
12	69M	8	D.E	3	2	III	1	0	M	C+M	-
13	58M	12	D.E	3	1	III	1	1	P	M	+
14	66M	11	D.E	3	1	III	1	1	M	C	-
15	66F	39	D.E	3	2	III	1	1	P	C	-
16	54F	16	D.E	3	3	III	1	0	W	C+M	-
17	52F	15	D.E	3	2	III	2	1	W	M	+
18	68M	12	D.E	3	1	III	U	U	n.a.	M	+
19	68M	26	D.E	3	2	III	1	2	W	M	-
20	68M	12	D.E	3	1	III	2	1	M	M	+
21	57F	14	D.E	3	2	III	2	2	W	M	+
22	61F	6	D.E	3	2	III	1	0	W	M	-
23	63M	28	D.O	3	3	III	3	1	M	C	+
24	47F	22	A	3	2	III	1	0	W	M	+
25	50M	29	A	3	2	III	1	1	P	C+M	+
26	68M	9	D.E	3	2	III	1	1	M	M	-
27	70F	4	D.E	3	2	III	1	0	W	M	-
28	56M	18	A	3	2	III	1	0	W	M	-
29	68M	9	D.E	4	0	III	U	U	U	M	+
30	68M	15	D.E	3	3	III	2	2	P	M	-
31	75M	6	D.E	4	3	IV	U	U	W	M	-
32	51F	9	D.E	4	2	IV	2	2	M	M	+
33	67M	2	D.E	4	2	IV	U	U	n.a.	M	-
34	55M	6	D.E	4	4	IV	U	U	M	M	-
35	51M	2	D.E	2	4	IV	0	0	M	M	+
36	69M	8	D.E	4	3	IV	U	U	W	C	+
37	62M	7	D.E	4	2	IV	1	1	W	M	-
38	45M	8	D.E	4	3	IV	1	0	W	M	+
39	46M	3	D.E	4	4	IV	2	1	M	M	+
40	52M	14	D.E	3	4	IV	1	1	M	C	+
41	72M	5	D.E	3	4	IV	2	2	M	M	-
42	53M	13	D.E	4	2	IV	1	0	M	M	+
43	76M	11	D.E	4	3	IV	1	1	M	M	+
44	64M	6	D.O	4	2	IV	1	1	M	M	-
45	51M	6	D.E	4	4	IV	U	U	W	M	-
46	58M	22	A	4	3	IV	U	U	M	C+M	-
47	69M	2	D.E	3	4	IV	2	1	W	C	+
48	57M	13	D.E	4	2	IV	2	2	M	M	-
49	72M	14	D.E	4	1	IV	1	1	W	C	-
50	60M	18	A	4	4	IV	2	1	M	N+C	+

All the samples except cases 18 (unknown) and 33 (adenosquamous carcinoma) were squamous cell carcinomas.

a) D.O; dead with other disease, A; alive D.E; dead with esophageal cancer

b) ly; grade of lymphatic invasion, U; unknown c) v; grade of venous invasion, U; unknown

d) differentiation, W; well differentiated, M; moderately differentiated, P; poorly differentiated, U; unknown, n.a.; not applicable

e) localization by immunostaining, M; membrane, C; cytoplasmic N; nuclear staining

### Immunohistochemical staining for cyclin D1

**Figure 2:**
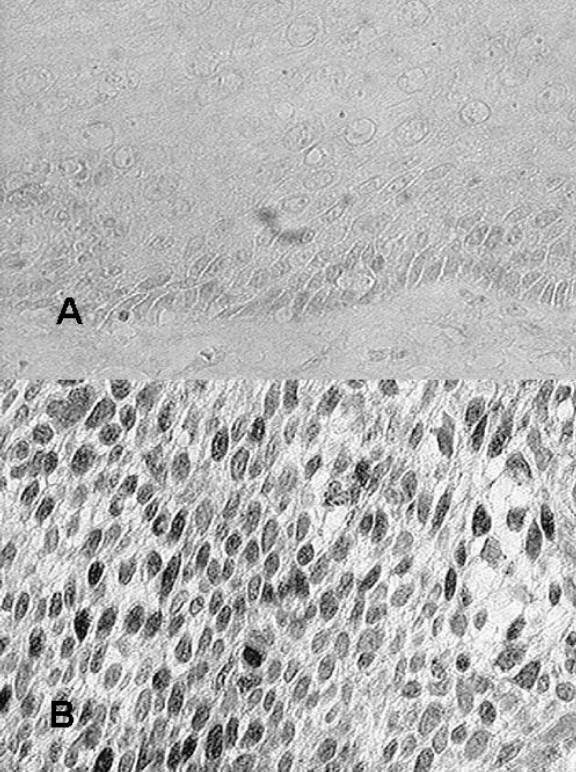
Immunohistochemical staining of cyclin D1 expression in normal and cancer tissues of the esophagus. In all normal esophageal mucosa, cyclin D1 staining was negative (a). In some cancer tissues, cyclin D1 was expressed at the nuclei (b).

We then analyzed the expression of cyclin D1, one of the possible downstream targets of the Wnt signal pathway, using immunohistochemistry. In all the normal esophageal mucosa, cyclin D1 staining was negative (Fig. 2a). Overexpression of cyclin D1 was observed in the tumor nuclei in 27 out of 50 (54%) patients (Fig 2b). All of the four cases that showed widespread nuclear β-catenin staining overexpressed cyclin D1. When these 4 cases were excluded, there was no cooperation between the staining pattern of β-catenin and cyclin D1 expression. No relationship was observed between the overexpression of cyclin D1 and age, sex, tumor size, stage, differentiation grade, lymph node metastasis, response to chemotherapy, or survival (Table 2).

### PCR and direct sequencing analysis of the β-catenin gene

To investigate the mechanism of β-catenin nuclear localization, we amplified the β-catenin gene by PCR using the primer pair flanking the entire exon 3. All four cases showed the single band of 502 bp, and none showed an aberrant PCR product within the amplified region. Then we determined the sequence of each band by direct sequencing. No mutational change was found in any of these cases (data not shown).

### PCR and direct sequencing analysis of the Axin gene

**Figure 3:**
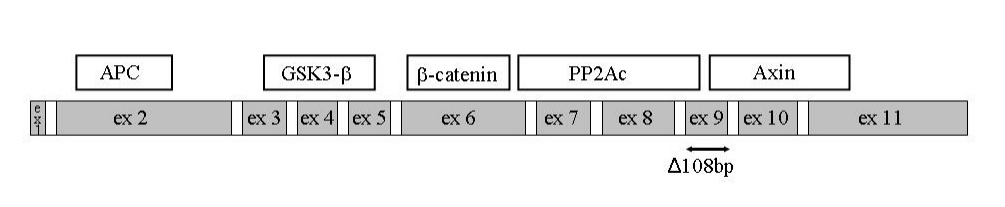
Genetic alterations of the Axin gene in esophageal squamous cell carcinoma. Splicing variant is indicated by two-headed arrows.

We then searched for mutational changes in the Axin gene. Axin gene mutation may have caused the abnormal distribution of β-catenin. We examined the Axin cDNA in four cases showing nuclear localization of β-catenin protein. Sequencing analysis of the Axin cDNA revealed a splicing variant (108 bp deletion, position 2302–2409) and a normal cDNA in two of the cases tested. We sequenced the cDNA from the normal mucosa in these cases, and the same variant was found. This deletion affected the whole exon 9 (Fig. 3). Thus neither β-catenin exon 3 nor Axin gene mutation was responsible for the aberrant localization of β-catenin in the 4 cases.

## Discussion

In this paper, we searched for changes in some of the proteins and genes involved in the Wnt signaling pathway in esophageal cancer. We identified abnormal nuclear accumulation of β-catenin protein in 4 (8%) out of the 50 esophageal cancer samples. The aberrant location of this protein was probably function suggested by the increased expression of cyclin D1 in all 4 of these cases. However, we were unable to find any genetic alterations responsible for this aberrant β-catenin distribution.

Ninomiya *et al*. have reported that overexpression of β-catenin was observed in 3 of 22 cases studied (13.6%). Although no mutation of the β-catenin gene was observed, the common silent mutation of the APC gene was found in all the cases [27]. De Castro *et al*. have reported that 18% cases of the esophageal squamous cell carcinoma showed overexpression of β-catenin in the cytoplasm and nuclei of tumor cells. They found no genetic alteration of the β-*catenin *gene [13]. We observed nuclear expression of β-catenin in 4 cases (8%). This is a frequency similar to those reported by other researchers. No mutational change in the β-*catenin *gene was found in any of the cases we studied, as reported by others [13].

In esophageal adenocarcinomas, Krishnadath *et al*. observed a correlation between reduced expression of β-catenin and poor prognosis [28]. Furthermore, in esophageal adenocarcinomas, the nucleus was stained in some tumors by β-catenin immunostaining. Lin et al. have also reported immunohistochemical data on β-catenin in esophageal cancer but did not find correlation with malignant behavior of the tumor [29]. In another study β-catenin and cyclin D1 expression was correlated with survival of esophageal cancer patients [30]. In other series of esophageal cancers, no mutation in the mutation cluster region of APC and exon3 of β-catenin genes was detected [7].

It has been reported that immunohistochemical examination of cyclin D1 expression may provide important prognostic information for esophageal cancer [31]. Takeuchi *et al*. have reported that the overexpression of cyclin D1 may be a useful prognostic indicator in patients with squamous cell carcinomas of the esophagus [32]. And Zhai *et al*. have reported that overexpression of cyclin D1 was highly associated with nuclear accumulation of β-catenin in ovarian endometrioid adenocarcinomas [33]. In our studies, no relationship was observed between cyclin D1 expression pattern and clinicopathological features. In accordance with the report by Zhai *et al*. all four cases in our study showing nuclear localization of β-catenin protein overexpressed cyclin D1.

A number of studies have shown that Axin is critical for mediating the down regulation of β-catenin [34,35]. One recent publication reported reduced axin repression in ESCC [36]. We looked for Axin gene mutations in esophageal squamous cell carcinoma, but found only a splicing variant.

In conclusion, the nuclear localization of β-catenin occurs in a fraction of esophageal carcinomas. All the cases showing nuclear localization of β-catenin protein overexpressed cyclin D1 which may have contributed to the oncogenesis of the esophageal carcinoma. The abnormal localization of β-catenin apparently did not result from the genetic alterations of either the β-catenin or Axin gene.

## Competing interests

The author(s) declare that they have no competing interests.

## Authors' contributions

YF and JK planned the experiment. JK performed most of the experiments with the help and supervision of NH and HI. TN, YS, YT HS, NS, MK, and YK provided the surgical materials. JK and YF wrote the paper.

All authors read and approved final version of manuscript.
